# Determining Hydroxyapatite Filling Volume for the Treatment of Post-Extraction Alveoli Based on Measurements of Alveolar Volume in Relation to the Body Weight of Dogs

**DOI:** 10.3390/vetsci11120633

**Published:** 2024-12-07

**Authors:** Anna Misztal-Kunecka, Przemysław Prządka, Maja Jeż, Stanisław Dzimira

**Affiliations:** 1 Veterinary Clinic DentalVet Anna Misztal-Kunecka, 71-795 Szczecin, Poland; 2 Department and Clinic of Surgery, Faculty of Veterinary Medicine, Wroclaw University of Environmental and Life Sciences, 50-375 Wroclaw, Poland; przemyslaw.przadka@upwr.edu.pl; 3 The Clinical Research Support Centre of the Pomeranian Medical University in Szczecin, 70-204 Szczecin, Poland; maja.jez@pum.edu.pl; 4 Department of Pathology, Division of Pathomorphology and Veterinary Forensics, Faculty of Veterinary Medicine, Wroclaw University of Environmental and Life Sciences, 50-375 Wroclaw, Poland

**Keywords:** alveolar volume, hydroxyapatite volume, dog, tooth size

## Abstract

Filling post-extraction alveoli with hydroxyapatite-based materials is becoming an increasingly common procedure in veterinary dentistry. In dogs, tooth roots vary in structure depending on the weight of the dog, but data on tooth length and volume have not yet been described. This study aimed to establish reference data on tooth root length and post-extraction alveolar volume for mature maxillary and mandibular incisors and canines in dogs. We determined the mean length and volume of these teeth in dogs in the weight ranges of 1–5 kg, 5–10 kg, 10–20 kg, and over 20 kg. The obtained values given showed a correlation between tooth length and alveolar volume in a specific weight range. A review of the commercially available hydroxyapatite-based bone substitute materials was then conducted. A table is presented which shows how to calculate the volume of bone substitute material required to fill a post-extraction alveolus with a given material. Statistics were used to assess significant differences between the mass of the bone substitute product used (μL) for specific weight ranges and to demonstrate the correlation between tooth length and alveolar volume for a specific weight range. The data obtained in this study can serve as reference values for tooth crown length and alveolar volume, allowing operators to plan a specific volume of bone substitute material for filling post-extraction alveoli. This research is interesting because it shows that the weight of an animal is an important aspect in planning the amount of bone substitute material for tooth extraction. In clinical work, it is much easier to weigh an animal than it is to make calculations based on the length of the tooth root.

## Simple Summary

This study aimed to establish reference data on tooth root length and post-extraction alveolar volume for mature maxillary and mandibular incisors and canines in dogs. We determined the mean length and volume of these teeth in dogs in the weight ranges of 1–5 kg, 5–10 kg, 10–20 kg, and over 20 kg. The obtained values given showed a correlation between tooth length and alveolar volume in a specific weight range. The data obtained in the study can serve as reference values for tooth crown length and alveolar volume, allowing operators to plan a specific volume of bone substitute material for filling post-extraction alveoli.

## 1. Introduction

Research on critical size defects (CSDs) in both long bones, as well as mandible and jawbone cavities, began more than 25 years ago. This term originally referred to bone wounds that did not heal naturally throughout the life of an animal. Later, cavities for which spontaneous healing comprise less than 10% of bone regeneration in the first year were considered to be CSDs. However, the remainder of the defect consists of fibrous tissue [1,2]. Other studies and analyses of the literature gave rise to the current thinking about bone repair processes and guided bone regeneration intended to provide the expected healing results [3]. Then, autogenous, isogenic, xenogenic, and alloplastic materials emerged, and many scientific articles are still being published today about their properties, disadvantages, and advantages. One of the most studied and popular alloplastic materials is hydroxyapatite. It is a substance that has been successfully used to regenerate bone not only in the alveolar process, but also in long bones. It is often employed in medical and veterinary fields, such as dentistry, orthopaedics, neurosurgery, traumatology, and plastic surgery [4,5]. It is a mineral from the calcium phosphate group, which occurs naturally in nature and is of great interest in the treatment of bone defects due to its specific properties [6]. It is one of the most valued bone fillers due to its biocompatibility, lack of carcinogenic properties, and high resistance to sterilisation processes. Hydroxyapatite in its porous form can not only serve as a bone filler but can also be used as a carrier for active substances such as antibiotics [7,8].

The available literature does not address the volume of either the teeth themselves in dogs or the volume of the alveoli in dogs. Due to the very large variation in the size of veterinary patients, the use of bone substitute materials designed for humans can be a challenge for surgeons. First, clinicians must determine how much implant material should be prepared in order to fill the alveolus tightly. Notably, veterinary patients, in addition to their weight differences, are also characterised by variations in cranial structure.

Differences in cranial structure, particularly with brachycephalic patients in mind, often cause tooth crowding and misalignment, resulting in occlusal conflicts. However, they do not cause differences in tooth structure, which is the case in human dentistry [9]. The second question that often arises when planning a procedure is which bone graft material to choose, considering not only its regenerative properties but also the simplicity of the procedure, the lack of additional implant equipment, and the physical and mechanical properties of the material in question.

Therefore, the aim of this study was to establish reference values for tooth root length and post-extraction alveolar volume and to analyse hydroxyapatite-based bone substitute materials to help surgeons accurately plan this part of the dental procedure on dogs.

## 2. Materials and Methods

### 2.1. Subjects

The first part of this study used a total of 600 teeth from dogs aged from 3 to 12 years with a weight range of 1 to 50 kg. The teeth were surgically extracted for periodontal reasons (vertical alveolar bone recession, grade II or III mobility, and lesions of the root apex that did not induce apical lysis). They were then thoroughly cleaned with a toothbrush and running water to remove anything adhering to the tooth surface and placed in 5.2% sodium hypochlorite solution for two minutes and stored in a container of 10% formalin until the measurements were taken. The containers were labelled with a description of the dog’s weight and the type of the extracted tooth. Teeth retrieved from brachycephalic dogs were excluded from the study due to their different anatomical structure. The criteria for exclusion from this study included having a severe root deformity, atypical root structure, non-closed root apex, root canal treatment and root hypercementosis; fillings altering the structure within the tooth neck; fractures within the tooth neck and root; and malocclusion. Teeth with a fractured crown were also included in this study as measurements were taken from the height of the tooth neck to the root, so the tooth crown was not assessed in this study. No division was made according to the sex of the animal.

All the procedures were carried out in accordance with the law and international recommendations for best practice in veterinary clinical care in all cases and in accordance with Polish regulations (Art. 1 (2) (1) Journal of Laws 2015 item 266). Consent from the Local Ethical Committee for Animal Experiments was not required. The owners agreed to include information about their animals in this study.

The dogs were divided into four weight groups: 1–5 kg, 5–10 kg, 10–20 kg, and over 20 kg. All the study dogs were rated using the BCS (Body Condition Score), and only the dogs with a BCS of 4 or 5 were eligible for this study. In each weight group, the extracted teeth were divided into 3 subgroups: group 1: I and II maxillary and mandibular incisors (long, thin teeth, usually without curved roots); group 2: III maxillary incisors (teeth with curved roots, often quite long and thick, with a structure resembling a miniature fang); and group 3: maxillary and mandibular fangs. Once a sufficient number of extracted teeth had been collected, they were divided into groups, with 50 teeth in each of the specific weight groups.

### 2.2. Methods

**Figure 1:**
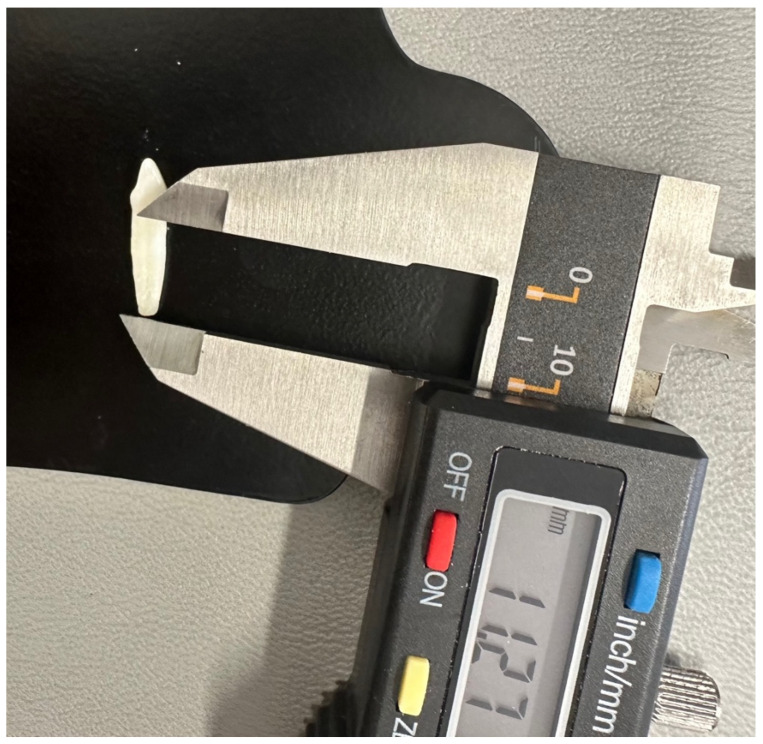
The length measurement of the maxillary second incisor.

**Figure 2:**
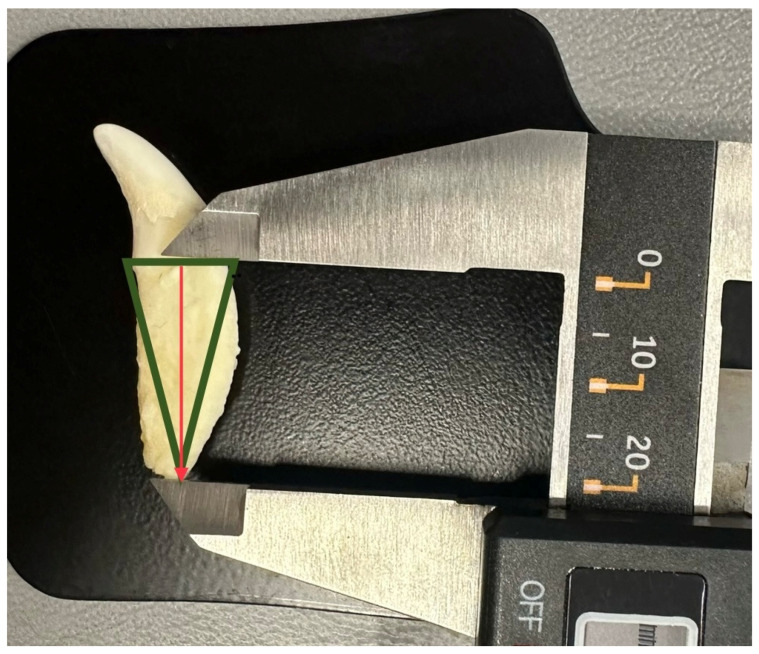
The measurement of the length of the canine. The green lines represent a triangle with the base of the length of the tooth neck and the apex at the longest end of the root apex. The red arrow shows the bisector of the angle, which determines the length of the tooth root.

A digital calliper with an accuracy of 0.01 mm was used to measure the length of the tooth root (Kormax 150 mm, SKC150, Manufacturer Kormax, Warsaw, Poland). When measuring the first and second incisors, the distance between the tooth neck and the apex of the root was determined as the tooth length (Figure 1). To measure the length of teeth with curvature (third incisor and fang), the bisector of the angle with its origin at the apex of the root and its end at the base of the triangle was determined, with a line drawn through the cervical part of the tooth (Figure 2).

Dental silicone (Elite HD+ Regular Normal, Zhermack, Badia Polesine, Italy) was used to measure the volume of the post-extraction alveoli. The synergistic action of the formulation’s nanotechnology and the immediate hydrophilic effect enable the precise reproduction of all the details. We chose to use this material because of its so-called ‘shape memory’, which means that even after a tooth has been extracted, the silicone returns to the shape in which it polymerised. The material is also characterised by a lack of reaction to physical factors, such as humidity, light and temperature.

**Figure 3:**
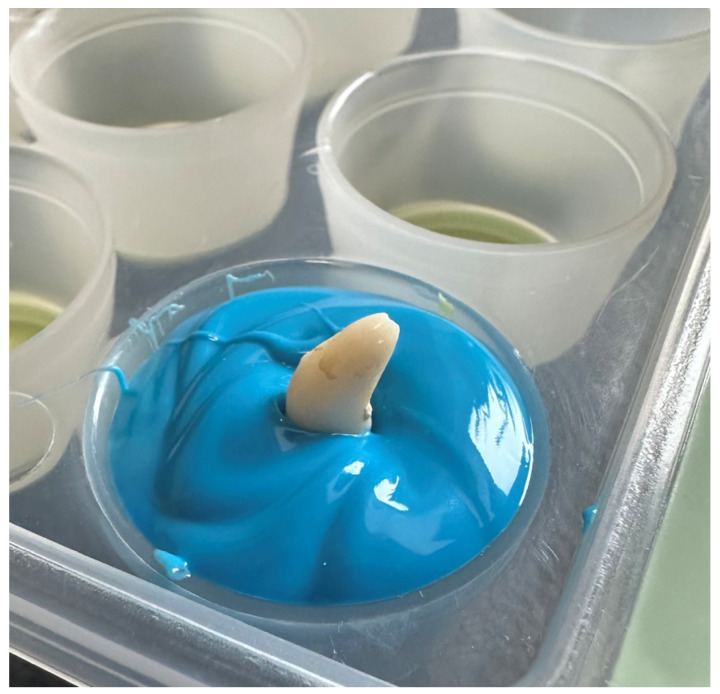
A tooth immersed in dental silicone while waiting for the material to polymerise.

**Figure 4:**
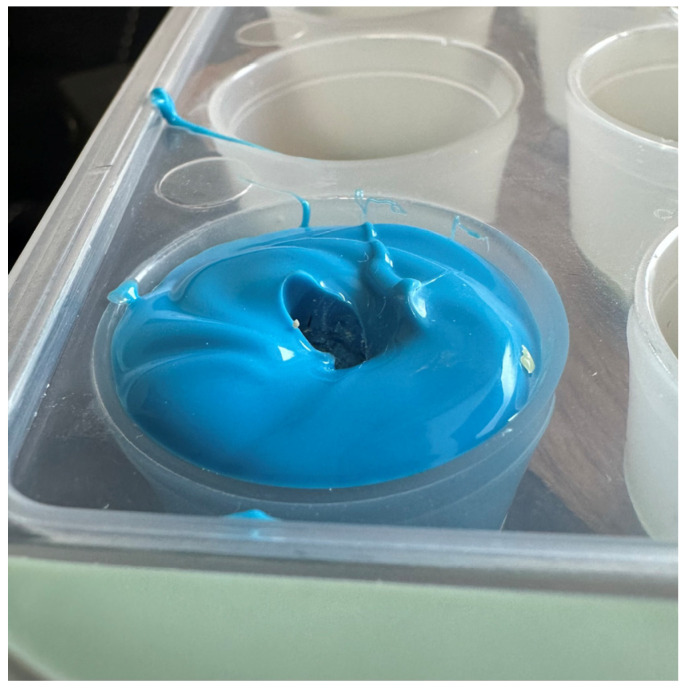
Tooth impression with accurate representation of anatomical structure prepared for filling with water to calculate tooth volume.

The layers of silicone were mixed according to the manufacturer’s recommendations using a dedicated mixing tip. Using an impression mass gun, the cavities in the medical tray, which had previously been ascribed to a specific weight group and tooth structure, were filled with silicone (Figure 3). After waiting the time recommended by the manufacturer (2 min 30 s), the teeth were immersed up to the height of the dental neck. After the polymerisation of the material within the time recommended by the manufacturer (3 min 30 s), the teeth were removed from the silicone mass, and an accurate impression of the alveolus was obtained. Then, using automatic pipettes with an accuracy of 20–100 µL, a volume of fluid was measured to fill the imprint to the line representing the transition between the root and the tooth crown (Figure 4). The root volumes of individual teeth were determined in this manner. The volume data and tooth lengths are included in Table 1.

## 3. Analysis of Hydroxyapatite Materials

An analysis of the four most popular hydroxyapatite-based materials available on the European market was carried out, focusing on their physical properties and respective implantation method. An analysis of the physical properties of the preparation and the possibility of tightly filling the alveolus as proposed by the manufacturer was performed. How much material would be needed to fill 1 cm^3^ of an alveolus was calculated, taking into account the physical properties and commercially available packaging. The data provided by the manufacturers and data from the literature data on the material in question are analysed and summarised in Table 2.

### Statistical Analysis

Analyses were carried out using the Statistica 13 programme (Statsoft, Tulsa, OK, USA). The *Shapiro–Wilk test* was used to verify the normality of data distribution. To verify the homogeneity of variance, Levene’s test was performed. The significance of differences between the samples was determined via the Kruskal–Wallis test at a *p*-value < 0.05. Descriptive statistics, including the means and standard deviations, were used to describe each variable analysed in this study. Spearman’s rank correlation analysis was performed to determine the correlations. For the graphical presentation of the distribution of the data, we used box-and-whisker plots.

## 4. Results

A total of 600 teeth from canines in four weight ranges were analysed: 150 teeth extracted from dogs weighing 1–5 kg, 150 teeth from dogs weighing 5–10 kg, 150 teeth from dogs of weighing 10–20 kg, and 150 teeth from subjects weighing more than 20 kg. They were then divided into three subgroups related to anatomical structure to assess length and volume: 50 maxillary and mandibular incisors I and II; 50 of maxillary incisor III; and 50 maxillary and mandibular canines. Statistically significant differences (*p* < 0.05) were found between the values of the volume (μL) of bone substitute material required to fill the alveoli for the same type of tooth depending on weight (kg) (Table 1). Furthermore, Spearman’s rank correlation coefficients showed a significant relationship between the tooth length (mm)/volume (μL) of the alveoli for specific weight ranges for all the samples, as shown in Table 3.

**Figure 5:**
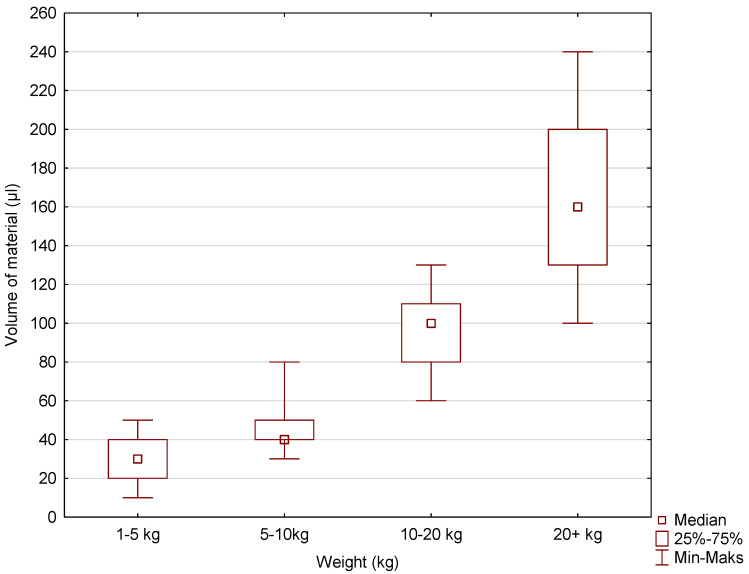
The box-and-whisker plot for incisors I and II for the variable volume of the alveolus (µL) vs. the cluster variable weight.

**Figure 6:**
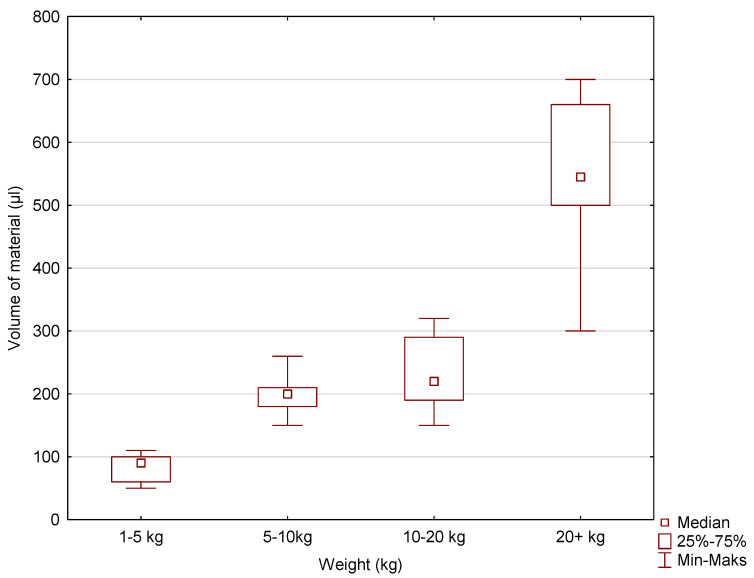
The box-and-plot for incisor III for the variable volume of alveolus (µL) vs. the cluster variable weight.

**Figure 7:**
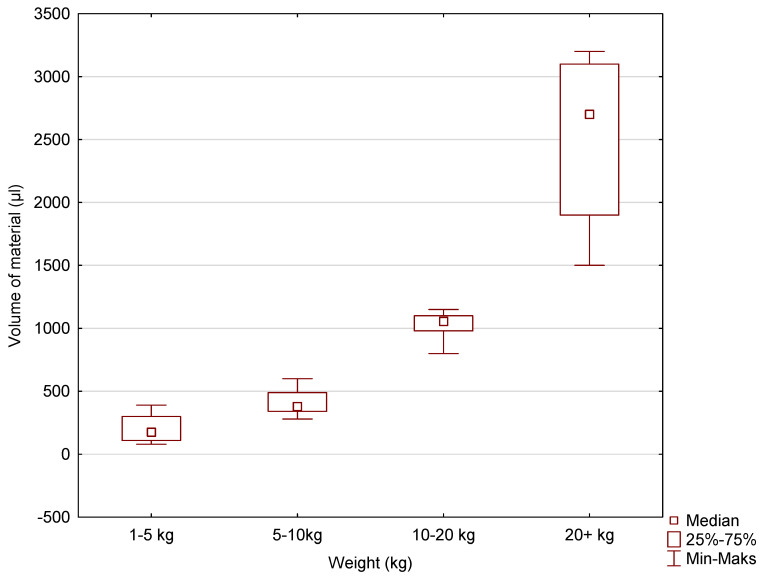
The box-and-whisker plot for the canine tooth for the variable volume of alveolus (µL) vs. the cluster variable weight.

Box-and-whisker plots were used to graphically represent the median, quartiles, and the minimum and maximum values of the data. The median (Me) alveolar volumes (μL) for incisors I and II in dogs in the 1–5 kg, 5–10 kg, 10–20 kg, and over 20 kg weight categories were 30, 40, 100, and 160, respectively. The Me values for maxillary incisor III were 90, 200, 220, and 545. The Me values for the canine were 175, 380, 1055, and 2700. The largest range of volume values was recorded for incisors I and II, maxillary incisor III, and the canines in the over 20 kg weight category (Figure 5, Figure 6 and Figure 7).

**Figure 8:**
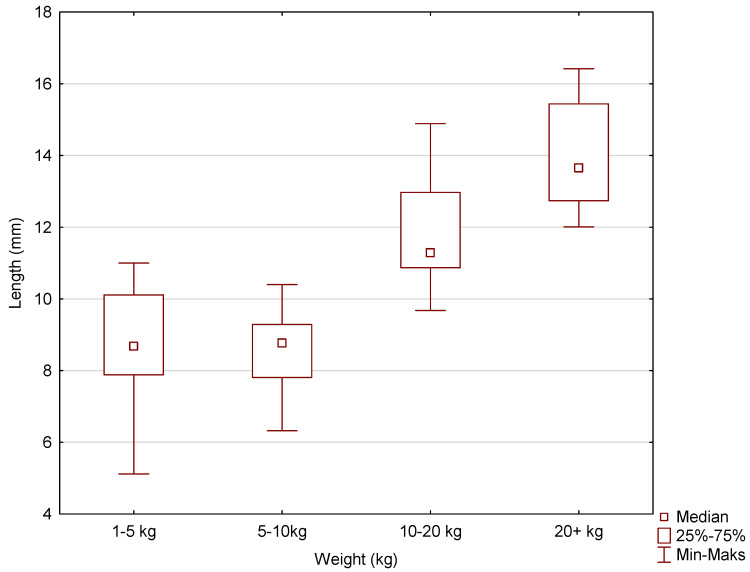
The box-and-whisker plot for incisors I and II for the variable length (mm) vs. the cluster variable weight.

**Figure 9:**
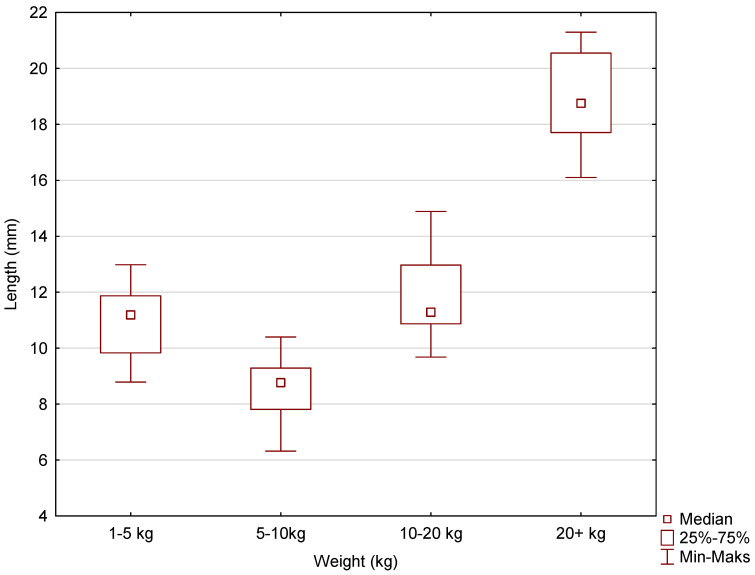
The box-and-whisker plot for incisor III for the variable length (mm) vs. the cluster variable weight.

**Figure 10:**
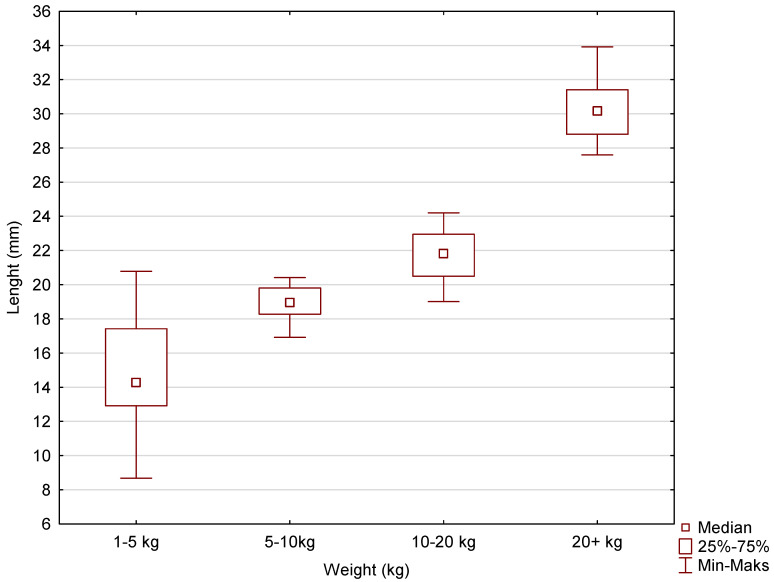
The box-and-whisker plot for the canine for the variable length (mm) vs. the cluster variable weight.

The average root lengths of maxillary incisors I and II were 8.65 ± 1.59, 8.53 ± 1.01, 11.85 ± 1.40, and 14.06 ± 1.41, respectively, for dogs in the following weight ranges: less than 5 kg, 5–10 kg, 10–20 kg, and more than 20 kg. The mean root lengths of maxillary incisor III were, respectively, 10.87 ± 1.22, 11.05 ± 0.59, 13.91 ± 1.74, and 18.90 ± 1.56. The mean canine root lengths were 14.90 ± 3.35, 19.02 ± 0.85, 21.64 ± 1.51, and 30.09 ± 1.56 for the patients in the weight range studied, i.e., less than 5 kg, 5–10 kg, 10–20 kg, and more than 20 kg. Box-and-whisker plots were used to graphically represent the median, quartiles, and minimum and maximum values of the data. The median (Me) lengths (mm) of the animals’ teeth for incisors I and II in the weight categories 1–5 kg, 5–10 kg, 10–20 kg, and 20+ kg were, respectively, 8.68, 8.77, 11.29, and 13.66. The Me values for incisor III were 11.19, 11.03, 14.00, and 18.76. The Me values for the canines were 14.27, 18.95, 21.82, and 30.16 (Figure 8, Figure 9 and Figure 10). The largest differences in the spread of tooth root length data were recorded for incisors I and II in the 1–5 kg weight group and, for incisor II, in the 10–20 kg weight group and also the canines in the 1–5 kg weight group.

There was a 38% difference recorded in the length of incisors I and II in the 1–5 kg and over 20 kg weight ranges, while the difference in alveolar volume was nearly 83% for the same teeth in different weight ranges. The difference in length of incisor III between the lowest and highest weight ranges was 43%, and the difference in volume was 83%. The largest differences in length and volume recorded for the canines in the weight ranges 1–5 kg and above 20 kg were 51% and 92%, respectively.

Four of the most popular hydroxyapatite-based bone graft materials were analysed. Granules are the most common form of bone graft substitutes (Adbone^®^, SinossGraft^®^). The other two materials come in the forms of hard, cylindrical pellets (PerOssal^®^) or cylinder/block pellets (FlexiOss^®^Vet). Only one of the materials shown is registered for use in animals (FlexiOss^®^Vet); the other preparations are used in human medicine (dentistry, orthopaedia, and traumatology). The FlexiOss^®^Vet and PerOssal^®^ preparations allow the operator to accurately calculate the amount of material necessary of material to fill the alveolus; their quantity is given in cm^3^. The other materials are given in weight grammage, so it is not possible to calculate the amount of material needed to fill the alveolus.

**Figure 11:**
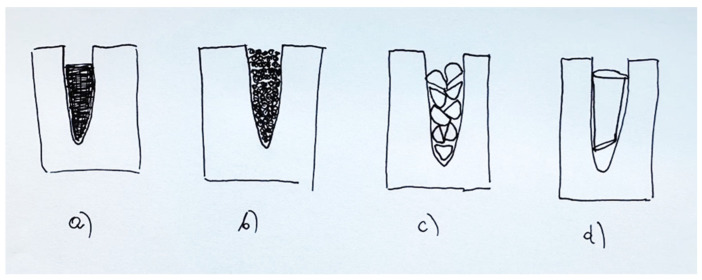
A schematic diagram showing the filling of alveoli with a bone substitute material (**a**) in the form of a cylindrical pellet after plasticity has been achieved (FlexiOssVet^®^), (**b**) in the form of granules (Adbone^®^, SinossGraft^®^), (**c**) in the form of cylindrical pellets (PerOssal^®^), and (**d**) in the form of a block/cylinder (Adbone^®^).

The FlexiOss^®^Vet material is the only material that increases in volume by approximately 30 percent within 48 h after implantation, which means that the surgeon can use correspondingly smaller volumes of the preparation to perform the procedure. The complete filling of the alveolus is possible with FlexiOss^®^Vet, Adbone^®^, and SinossGraft^®^. Of these three, FlexiOsst^®^Vet is the best filling material, as it is malleable after soaking in saline, blood, or antibiotics and can be cut and shaped to suit the dimensions of the alveolus. PerOssal^®^ and Adbone^®^ cylinders cannot fill the alveolus tightly because of their specific shape, even if the amount of material required for filling is calculated (Figure 11). An additional property of FlexiOss^®^Vet and PerOssal^®^ is that they can carry antibiotics, making general antibiotic therapy unnecessary, even in cases of severe alveolar bone inflammation.

## 5. Discussion

The aim of this study was to obtain information on tooth root length, alveolar volume, and the possibility of filling the alveolus with a bone substitute material. To date, there have been no publications on tooth length and volume in dogs. The existing scientific dentistry reports compare tooth structure and length between individuals from different continents and races, which is important for orthodontic treatment [14,15,16]. Other authors calculate tooth length based on modern cone beam computed tomography (CBCT) imaging techniques, which minimises the risk of error due to the one-dimensionality of the images and possible human error during X-ray imaging [17,18]. In the present study, we focused on the root length of teeth in vivo, as the teeth needed to be extracted for medical reasons and the alveoli filled with material in any case. Measuring an extracted tooth is easier for the surgeon and subject to less error than X-ray-based measurement. In the available literature, there have been attempts to compare the length from the crown to the root of teeth, but an algorithm can be used to calculate this length of the root without the need for X-rays or tooth extraction based on crown length alone. The difficulties encountered, including age-related crown wear, different biting patterns, tooth fractures, and behavioural problems affecting the shape of the tooth crown, meant that further studies were abandoned [19,20]. The reports in the literature agree that measurement with a digital calliper is sufficient to measure the root of a tooth after extraction, as proposed by Dashrath, K. et al. [21]. In the presented study, we also used a digital calliper as the measuring device. The literature review did not provide us with an answer to the question of the volume of post-extraction alveoli in either humans or dogs. The available literature only discusses the volume of the teeth themselves in the context of orthodontic treatment planning [22,23]. The robust increase in digitalisation makes it possible to calculate the root volume of individual teeth using CBCT imaging [24]. The volume of a tooth can also be calculated physically using the water displacement method by subtracting the initial water volume from the final volume after immersing the tooth in water in a graduated cylinder [25,26]. As we measured alveoli and not teeth, we modified the method to suit the purpose of this study. Dental silicone, before it was superseded by computerisation, was widely used to take impressions for prosthetic work not only in dentistry but also in other branches of prosthetics [27]. The ability to perfectly reproduce even the finest anatomical structures allowed the authors to accurately model the alveolus and delineate the line between the tooth neck and bone. We agree that this method appears to be superior to working on cadaveric teeth. Cadavers probably accurately represent alveoli, but anomalies, such as alveolar bone atrophy, pathological pockets, periapical lesions, and moist or dry tissue, could significantly negatively affect the results [28].

Although the presented study did not determine tooth lengths using diagnostic imaging, the box-and-whisker charts provide clinicians with the opportunity to estimate alveolar volume based on the length of a particular tooth type as long as it falls within the size range indicated on the chart. Given the data presented in the study, this is not the key aspect in planning the amount of bone substitute material to be used to fill the post-extraction alveolus, but rather, it is the weight of the animal.

A review of the data on bone graft materials did not provide us with information on why manufacturers choose to specify the amount of material in the package in grammes or cubic centimetres. Stating the amount of preparation in units of volume makes the process easier for the surgeon. Although the literature on both humans and animals does not mention the volume of the alveolus, it is possible to estimate the amount of preparation needed for the procedure based on the volume of the tooth root, and there are plenty of such data when it comes to human dentistry, including a geographical breakdown [5,16,29]. No article discussing either the volume of the alveolus or the tooth itself in dogs was found.

The available literature does not address the difficulties the surgeon may encounter when filling post-extraction alveoli with the bone substitute materials available on the market due to their shape. After tooth extraction, the natural vascular reflex is to fill the alveolus with blood and then produce a clot, which is the base for the granulation tissue and will initiate the healing of the alveolus. The next step is to heal the alveolus. In the context of alveolar wound healing, generating a cell contraction force in the post-extraction wound is of great importance, as it stabilises the wound and serves as a matrix for bone formation [30,31]. As the balance depends on the relative mass of the two edges, the buccal wall will give way under stress due to the structure and begin to undergo gradual lysis [32]. Therefore, in both human and veterinary dentistry, there is a lot of debate about securing the alveolar ridge with bone replacement materials, rather than leaving it to the primary healing process.

Granule implantation involves them being partially pushed outwards via the pressure of outflowing blood. Therefore, it can be difficult, or often even impossible, for an inexperienced surgeon to tightly fill the alveolar cavity completely with granules. After implantation, it is necessary to close the wound tightly with a gingival flap so that the bone substitute material does not move or is not completely removed from the implant site. Clinically, more convenient materials are those that mimic the shape of the alveolus, e.g., cylindrical pellets, cylindrical moulds and cylinders/blocks. Thanks to their shape, they are much easier to embed in the alveolus and are not pushed out by flowing blood. The shape of these moulds is standard, making it difficult to fill the alveolus completely. Only the FlexiOss formulation becomes malleable due to its curdlan content, so it seems to be the most suitable.

## 6. Conclusions

A correlation was shown between tooth root length and alveolar volume for dogs in the weight ranges 1–5 kg, 5–10 kg, 10–20 kg and over 20 kg. This study shows that the weight of the animal is an important aspect in planning the amount of bone substitute material needed to fill post-extraction alveoli. The results of this study can serve as reference values for anthropometric measurements in dogs.

An analysis of hydroxyapatite-based bone substitute materials showed that the tight filling of the alveoli would be easiest with a preparation method whose combination with curdlan provides plasticity to the implant material.

## Figures and Tables

**Table 1:** A comparison of tooth lengths and alveolar volumes for the different tooth types in the weight groups used. *Kruskal–Wallis test* (*n* = 50). The results are expressed as the mean ± SD; *n* = 50.

	Incisors I and II		Incisor III		Canine	
	mm	µL	mm	µL	mm	µL
1–5 kg	8.65 ± 1.59	28.80 ± 10.91	10.87 ± 1.22	83.80 ± 22.76	14.90 ± 3.35	193.60 ± 94.93
5–10 kg	8.53 ± 1.01	45.60 ± 10.72	11.05 ± 0.59	196.40 ± 26.17	19.02 ± 0.85	408.20 ± 97.41
10–20 kg	11.85 ± 1.40	97.20 ± 18.19	13.91 ± 1.74	230.00 ± 50.59	21.64 ± 1.51	1018.20 ± 102.61
20+ kg	14.06 ± 1.41	168.80 ± 66.61	18.90 ± 1.56	551.80 ± 119.30	30.09 ± 1.56	2510.00 ± 591.18
*Kruskal–Wallis test*	H = 152.73 *p* < 0.05	H = 173.82 *p* < 0.05	H = 162.92 *p* < 0.05	H = 170.42 *p* < 0.05	H = 166.97 *p* < 0.05	H = 182.19 *p* < 0.05

**Table 2:** Comparison of hydroxyapatite-based bone substitute materials.

Tested Material	Manufacturer, Country of Origin	Composition	Form of Material	Available Packaging	Amount of Material Required to Fill 1 cm^3^ of Alveolus	Possibility of Tight Filling of the Alveolus	Possibility of Filling Alveoli with the Missing or Damaged Alveolar Wall	Intended Purpose
FlexiOss^®^ Vet [10]	Medical Inventi, Poland	Hydroxyapatite polymer + curdlan	Cylindrical pellet	Single-packed pellets: 0.2 cm^3^, 0.5 cm^3^, 1 cm^3^, 3 cm^3^, 5 cm^3^	0.7 cm^3^ (1 pack of 0.2 cm^3^ + 1 pack of 0.5 cm^3^) (material expands and increases in volume by 30% within 48 h after implantation)	Yes, the material becomes malleable after soaking and can be adapted to the alveolus	Expansion and YES	Dentistry, veterinary orthopaedics (equivalent in human dentistry, FlexiOss^®^Dent, in orthopaedics, traumatology and neurosurgery, FlexiOss^®^)
PerOssal^®^ [11]	Osartis GmbH, Germany	51.5% hydroxyapatite + 48.55% calcium sulphate	Cylindrical pellets	6 pellets = total 1.5 cm^3^, 12 pellets = 3 cm^3^, 50 pellets = 12.5 cm^3^	4 pellets of 0.25 cc (1 pack of 6 pellets)	NO	NO	Human orthopaedics
Adbone^®^TCP [12]	Medbon^®^ Biomaterials, Portugal	75% hydroxyapatite + 25% beta TCP	Granulate sizes 0.1–0.5 mm, 0.5–1 mm, 1–2 mm, blocks, cylinders	Granules 0.5 g and 1 g, blocks 5 × 10 × 15 mm, 8 × 8 × 20 mm, 15 × 15 × 20 mm, cylinders 8 × 8 × 20 mm	1 block 5.10 × 15 mm, granules in; no manufacturer data available	Granules—yes; cylinders, blocks—no	Blocks, cylinders—yes; granules—no	Orthopaedics, dentistry, neurosurgery, traumatology, human aesthetic medicine
SinossGraft^®^ [13]	Novadento, Germany	25% TCP + 75% hydroxyapatite	granules	Granules 0.5–1 mm, 1–2 mm 2 g, packs	No manufacturer data available	YES	NO	Human dentistry

**Table 3:** Spearman’s rank correlation coefficients indicating the relationship between the length of the teeth (mm) and the volume (μL) of the alveoli within certain weight ranges.

Type of Tooth		Spearman’s Rank Correlation
1–5 kg	Incisors I and II	0.768
	Incisor III	0.888
	Canine	0.858
5–10 kg	Incisors I and II	0.743
	Incisor III	0.606
	Canine	0.911
10–20 kg	Incisors I and II	0.416
	Incisor III	0.748
	Canine	0.968
20+ kg	Incisors I and II	0.867
	Incisor III	0.906
	Canine	0.904

## Data Availability

All data are contained within this article.
